# Radiomics analysis of early pregnancy ultrasound images to predict viability at the end of first trimester

**DOI:** 10.1038/s41598-026-35158-5

**Published:** 2026-01-28

**Authors:** Sughashini Murugesu, Kristofer Linton-Reid, Jennifer Barcroft, Margaret Pikovsky, Srdjan Saso, Eric O. Aboagye, Tom Bourne

**Affiliations:** 1 Queen Charlotte’s and Chelsea Hospital, Imperial College, London, W12 0HS UK; 2 Department of Metabolism, Digestion and Reproduction, Imperial College London, Du Cane Road, London, W12 0NN UK; 3 Department of Cancer and Surgery, Imperial College London, London, UK; 4 Research and Innovation Centre, African Institute for Mathematical Sciences, Kigali, Rwanda

**Keywords:** Ultrasonography, Pregnancy outcome

## Abstract

To determine whether there are radiomic ultrasound features of early pregnancy when viability is unknown, which in combination with clinical features, may predict subsequent loss. Multi-centre retrospective cohort study, which included 500 cases of pregnancies of unknown viability (PUV) collected from January 2021 to January 2023. Longitudinal ultrasound images were identified from Queen Charlotte’s and Chelsea Hospital (QCCH), London (*n* = 400, split 8:2 for training and validation) and St Mary’s Hospital (SMH), London (test data set *n* = 100). Images were extracted and segmented to include firstly the gestation sac and secondly the sac endometrial border. A segmentation model was developed using a deep learning (DL) model (multi-task nnUNet v2) and standard Dice Coefficient (DICE) was used to measure performance. A prediction model, using clinical and radiomic features, was developed by comparing several machine learning (ML) methods. The area under the ROC curve (AUC), F1-score, and recall were used to assess model performance. The QCCH and SMH data sets were in the majority well matched and consisted of 53.3% and 53.0% miscarriage cases by the end of first trimester, respectively. The DL segmentation model for gestation sac achieved a mean DICE score of 0.950 and 0.940 in the training and test data sets respectively. The segmentation model for the sac endometrial border achieved a mean DICE score of 0.917 (QCCH) and 0.922 (SMH). The best performing PUV outcome classification model (XGBoost and LASSO) for predicting miscarriage (PUVPS model); achieved an AUC of 1.00 (F1-score 1.00), 0.92 (F1-score 0.79) and 0.84 (F1-score 0.76) in the QCCH training, QCCH validation and SMH test set respectively. We have developed an end-to-end radiomics-based model to segment and predict early pregnancy outcomes. The main limitation of this study is its sample size, which can make a ML model prone to overfitting. This study sets the stage for future trials to prospectively evaluate the performance of the PUVPS model, in a large multi-centre cohort, which can then be used to help patients navigate the uncertainty of a PUV early pregnancy classification.

**Supplementary Information:**

The online version contains supplementary material available at 10.1038/s41598-026-35158-5.

## Introduction

 Miscarriage is the most common pregnancy complication causing significant psychological and physical morbidity to women and their partners^1,2^. Miscarriage can occur at any stage in the first trimester, affecting 1 in 4 women. Despite advances in pregnancy research, the mechanisms behind miscarriage remain poorly understood. It is known that a proportion of miscarriage is due to aneuploidy^3^. It is also known that successful implantation, decidualisation and placentation in the first trimester are critical to pregnancy success and that the progression of a pregnancy is determined from this time^4^. Abnormalities during these early stages have been implicated in a number of adverse pregnancy outcomes, such as miscarriage and pre-eclampsia^4^.

Ultrasound is the imaging modality used to diagnose miscarriage in clinical practice. Diagnosis of miscarriage is determined by either an ultrasound finding of fetal pole crown rump length (CRL) >/=7 mm and no heartbeat, or by assessing the rate of early pregnancy growth between two time points depending on the initial size of pregnancy of unknown viability (PUV) as per NICE guidelines^5^. Clinicians use ultrasound indicators for patient counselling, and in some cases to decide upon the frequency of follow-up ultrasound scans. Bottomley et al. 2013 published a scoring system to predict pregnancy viability with the following ultrasound variables: mean gestation sac diameter, mean yolk sac diameter and fetal heartbeat^6^. These data can be used to counsel patients about individual outcome, either to reassure or identify pregnant women with an elevated risk of pregnancy loss. Recent work has demonstrated that artificial intelligence (AI) can enhance early pregnancy ultrasound interpretation. Liu et al. (2025) applied an AI-based approach to automate nuchal translucency assessment in first-trimester ultrasound, showing that machine learning can improve measurement consistency and prediction accuracy^7^.

This study aims to identify radiomic ultrasound features from images derived from pregnancies classified as a PUV, which may predict subsequent loss. Radiomic analysis quantifies high-dimensional tissue features that cannot be observed by direct human eye analysis, and has shown potential in precision medicine, particularly in the oncological field^8,9^. Radiomics offers a quantitative method to assess heterogeneity between images, which can be more stable compared to subjective assessment. We propose that there may be radiomic features on early ultrasound scan (USS), with an overall finding of pregnancy of unknown viability, that may strengthen the prediction of viability. This in turn may help counsel women at a difficult point of uncertainty. To the best of our knowledge, there have been no previous studies apply radiomic analysis to PUV first trimester ultrasound images.

## Materials and methods

### Study population

This is a retrospective study of women presenting to the early pregnancy unit, with an ultrasound diagnosis of ‘pregnancy of unknown viability’ (PUV). From January 2021 to January 2023, pregnant women who underwent as ultrasound and were diagnosed with PUV, were retrospectively collated from the Astraia system in Queen Charlotte’s and Chelsea Hospital (QCCH) (London, UK) and St Mary’s Hospital (SMH) (London, UK).

A PUV is defined as the visualisation of one of the following on transvaginal ultrasonography (TVS): an empty intrauterine sac ≤ 25 mm; visualisation of a fetal pole ≤ 7 mm without demonstration of embryonic cardiac activity; a single intrauterine gestational sac with MSD < 25 mm and a visible yolk sac but no visible embryonic pole. These findings can indicate a normal early pregnancy of approximately 4-6weeks gestational age or a failing pregnancy leading to miscarriage.

The inclusion criteria were (1) singleton pregnancies presenting to the early pregnancy unit (2) undergoing a transvaginal ultrasound with a diagnosis of intrauterine pregnancy of unknown viability (3) included cases needed to have a longitudinal cross-section image of the uterus containing the pregnancy tissue.

The exclusion criteria included (1) patients with callipers or annotation on the longitudinal ultrasound image of the pregnancy within the uterus (2) those who were not followed up within the unit and thus the outcome at the first trimester dating scan (11 + 2 to 14 + 1 weeks gestation) was unknown.

### Outcomes

The primary outcome of the classification model was the pregnancy status at the end of the first trimester, a routine check-point in antenatal care, where an ultrasound is performed to determine viability. Miscarriage before or viability confirmed at this gestation were the two potential outcomes to be predicted with this model.

### Statistical analysis

To assess the model’s performance for accurate first trimester viability prediction, we used the F1-Score (harmonic mean of precision and recall), precision and recall (sensitivity). For clinical relevance we also calculated Area under the ROC curve (AUC), and specificity. Quantitative statistics were presented as median and interquartile range (IQR). Continuous variables were compared using the Mann-Whitney U test in non-normally distributed data and the independent t-test in normally distributed data, and categorical variables were compared using the Fisher’s exact test. The analysis was carried out using R.

### Radiological evaluation and clinical features

Radiological evaluation was recorded from the documentation within the ultrasound report, written by the clinician carrying out the ultrasound. All scans within these units are undertaken or supervised directly by expert clinicians with pelvic imaging experience of more than 1000 scans.

Data recorded included: Gestation Sac (GS) Mean Sac Diameter (MSD) measurement (mm), Sac Shape (irregular/regular), Crown Rump Length (CRL) measurement (mm), Yolk Sac presence (yes/no), presence of subchorionic haematoma, fibroids or ovarian cysts (yes/no).

Clinical features and patient demographic data recorded included: Maternal Age, Gravida, Parity, Previous Vaginal Delivery (number), Previous Caesarean Section Delivery (number), Gestation Age at time of USS by last menstrual period (LMP) (days), Bleeding Score at Presentation, Worst Bleeding Score in Pregnancy, Pain Score at Presentation, Worst Pain Score in Pregnancy, Pain in Pregnancy (yes/no). Only features that were routinely recorded in the early pregnancy unit were included to ensure usability of the final model. Maternal BMI^10,11^ and paternal age^12^ have been shown to have an impact on fertility and pregnancy outcomes, however were not consistently recorded and thus excluded.

To meet pre-processing requirements for machine learning, categorical data were converted to numeric. One-hot-encoding converted each level of each categorical feature into a new binary feature. Missing clinical data were assumed missing at random and non-dependent on outcome. Missing data was imputed using the multiple imputation with chained equations (MICE) package with default arguments in R. Highly correlated features were removed using the treatment_corr function, with a threshold of 0.85, removing one of each pair of correlated features (Pearson correlation for continuous and Spearman correlation for categorical features). Continuous features were standardized.

### Image segmentation

All ultrasound examinations were carried out using a standardised approach, using various ultrasound systems including: Voluson GE (E8, E9, E10) and Samsung (W10). 2D ultrasound, grayscale images without any callipers or annotation were used. For each case a single image was included: the sagittal transvaginal ultrasound view of the uterus containing pregnancy tissue, this image was retrieved from Astraia images for segmentation. Ultrasound images were acquired by multiple clinicians using a range of ultrasound systems as part of routine clinical care, providing a representative real-world dataset for radiomic analysis.

**Fig. 1:**
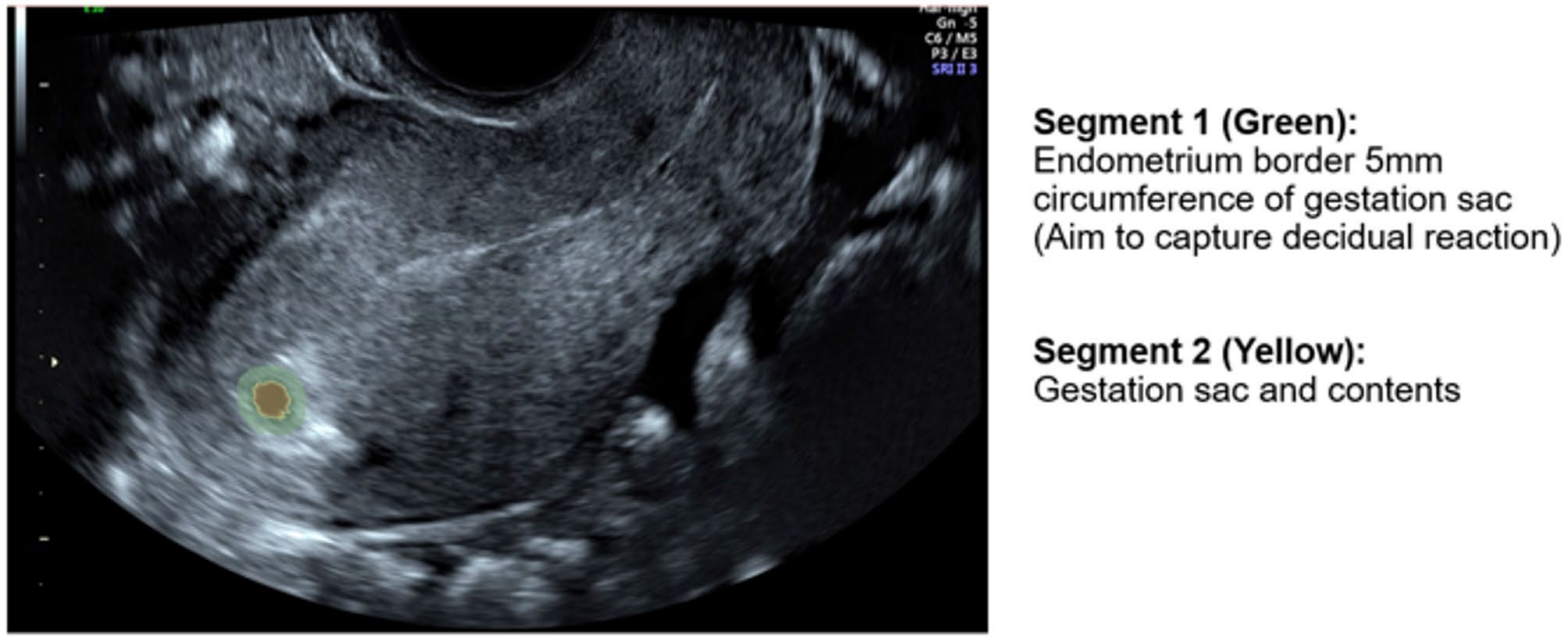
Example of Ultrasound Image with the two regions of interest segmented.

3D Slicer software (https://www.slicer.org/) was used for image segmentation. In order to reduce bias derived from the individual undertaking segmentation for the region of interest, the ‘threshold’ function was used to delineate the gestation sac from any surrounding trophoblast or endometrial tissue, and to define gestation sac and content correlating with a fetal pole or yolk sac (Segment 1, Fig. 1)). A fixed margin of 5 mm extending outward from the outer border of the gestational sac into the surrounding trophoblastic/endometrial tissue was segmented using the ‘margin’ function on the 3D slicer software (Segment 2, Fig. 1), with the aim to capture the decidual reaction and thus early trophoblastic tissue.

### Model development

**Fig. 2:**
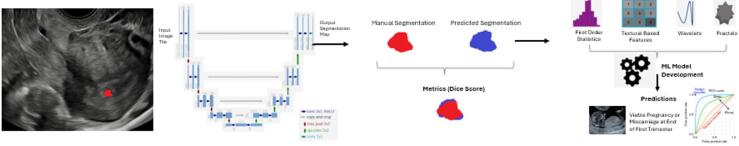
Model Development Flowchart: Deep Learning for Segmentation and Radiomics for Viability or Miscarriage Prediction.

To enable the development of an end-to-end classification model (Fig. 2), the first step involves the development of a segmentation model, to identify the regions of interest (gestation sac and gestation sac outer endometrial border 5 mm). The second step requires the development of a classification model to determine if the pregnancy is viable or non-viable at the end of the first trimester.The methods to develop this model follow an approach as previously published by this group^13^.

Multi-task nnUNet (v2), a deep learning model designed for medical image segmentation, was used to automate the segmentation of the outer gestation sac endometrial border segment and the gestation sac segment. Segmentation model development involved training on the QCCH development training dataset (320 cases) and the segmentation performance was evaluated on QCH validation (80 cases) and SMH test set (100 cases).

### Radiomic feature extraction

Radiomics features were extracted using TexLab 3.0, with various grey level binning ranges, to compute a total of 4435 radiomics-based features.

We used the Transparent Reporting of a Multi-variable Prediction Model for Individual Prognosis or Diagnosis (TRIPOD, https://www.tripod-statement.org/) guidelines for reporting the development and validation of the prediction models.

### Feature selection

In this study, conducted entirely in R, a variety of feature selection techniques were integrated to enhance the robustness and interpretability of our machine learning models.

Regularisation methods, such as LASSO and Elastic Net regression, were utilised via the glmnet package. These methods apply penalties to the coefficients of a linear model, thereby working to mitigate and reduce overfitting and leading to the selection of a subset of key features. LASSO employs an L1 penalty to force some coefficients to zero, while Elastic Net combines both L1 and L2 penalties, incorporating features of both ridge regression and LASSO.

Correlation-based methods including Pearson, Spearman’s, and Kendall’s rank correlation were also employed. Implemented using the ‘corr’ package in R, these methods select features based on their individual characteristics, with Pearson assuming linear relationships and Spearman’s and Kendall’s focusing on monotonic relationships.

Univariate logistic regression was another approach employed, using the glmpackage in R. This technique involved assessing each feature with the outcome variable and selecting those with a p value less than 0.05, adjusted for multiple comparisons using the Benjamini & Hochberg method.

Recursive Feature Elimination (RFE), implemented with the ‘rfeControl’ package in R and utilising a random forest model, was used to iteratively eliminate features based on their importance, employing crossvalidation to identify the most effective subset.

Mutual Information, was incorporated to evaluate the relevance and redundancy of features in predicting our target variable. The Boruta method was employed as a wrapper around a Random Forest classifier for iteratively removing less relevant features through statistical testing.

### Machine learning algorithms

Our classification models, all developed in R, utilised a diverse array of supervised algorithms. Logistic Regression, along with its regularised variations LASSO and Elastic Net, were key components in our modelling approach. Linear Support Vector Machines (L-SVM) were also employed for their ability to classify data by finding an optimal separating hyperplane. The K-Nearest Neighbours (KNN) algorithm was used for its simplicity and effectiveness, classifying data based on the proximity of points in the feature space. Ensemble decision-tree-based models like Random Forest (RF) and Extreme Gradient Boosting Machines (XGB) were selected for their robustness and accuracy in handling complex classification tasks. Partial Least Squares (PLS) was particularly useful in scenarios where we dealt with a large number of features and significant collinearity among them. For certain classification tasks, a Single-Layer Feed-Forward Neural Network, implemented using the ‘nnet’ package, was found to be effective. In addition, the Naïve-Bayes (NB) algorithm was employed for its probabilistic approach to classification, leveraging Bayes’ theorem and assuming conditional independence among features.

## Results

### Clinical characteristics of data

The QCCH development dataset consisted of 400 cases; the median age was 34 years old (IQR18-48) (Table 1). Within this cohort 187 (46.8%) went on to be viable and 213 (53.3%) had miscarried by the end of the first trimester. This dataset was randomly split 80:20 to comprise the training and validation data sets. The SMH dataset provided an external test dataset, which consisted of 100 cases; the median age was 33 years old (IQR 18–45) (Table 1). Within this cohort 53 (53%) went on to be viable and 47 (47%) had miscarried by the end of the first trimester.

**Table 1:** Cohort descriptive Statistics.

Parameter	Combined Training & Validation data (*n*=400)	Test data (*n*=100)	*P*-value
**Median Maternal Age (**Range)	34 (18–48)	33 (18–45)	0.1642
**Maternal Ethnicity** **-**Asian and Asian British -Black, Black British, Caribbean or African -Mixed or multiple ethnic groups -White -Other ethnic group -NR	92 34 22 181 69 2	22 15 3 31 29 0	0.2004
**Fibroids** -Yes -No -NR	16 379 5	4 95 1	1.0000
**Ovarian Cysts** -Yes -No -NR	10 385 5	5 94 1	0.1701
**Gravida** −1 −2 −3 −4 −5 −6 >6 -NR	103 96 82 41 36 15 11 16	20 25 22 13 8 5 4 3	0.2270
**Parity** −0 −1 −2 −3 −4 −5 ->5 -NR	191 125 46 16 4 1 2 15	44 32 12 7 1 1 0 3	0.3470
**Number of Previous Miscarriage/Termination of Pregnancy** −0 −1 −2 −3 ->3 -NR	170 112 54 33 15 16	41 30 13 7 6 3	0.7261
**Previous VD** −0 −1 −2 ->2 -NR	259 63 22 12 44	60 18 9 5 8	0.4667
**Previous CS** −0 −1 −2 ->2 -NR	284 56 13 3 44	72 12 5 3 8	1.0000
**Median Gestation of PUV by LMP** (days) (Range) **NR**	45 (8–129) 16	46 (28–86) 3	0.3010
**Median MSD (mm) (range)**	8.6 (1.6–31.4)	10.3 (2.3–26.9)	0.2379
**Gestation Sac Shape** -Regular -Irregular -NR	322 153 25	83 17 0	0.3226
**Yolk Sac** -yes -no	271 129	65 35	1.0000
**Fetal Pole** -yes -no	299 101	20 80	1.0000
**Median Fetal Pole CRL if present (mm)** (Range)	2.8 (1.0–6.4)	2.4 (1.1–6.1)	0.1494
**Subchorionic Haematoma** -yes -no	36 364	11 89	1.0000
**Bleeding Score at Presentation** −0 −1 −2 −3 −4 -NR	221 113 53 6 2 5	44 53 1 0 0 2	0.6120
**Worst Bleeding Score** −0 −1 −2 −3 −4 -NR	194 123 62 12 4 5	39 54 5 0 0 2	0.9763
**Worst Pain Score** −0 −1 −2 −3 −4 −5 −6 −7 −8 −9 −10 -NR	184 16 18 32 17 16 12 12 15 5 7 66	35 1 11 26 4 3 1 0 2 0 1 16	0.3390
**Pain at Presentation** -yes -no -NR	202 184 14	62 36 2	0.0544
**First Trimester Outcome** -VIUP -Miscarriage	187 213	53 47	0.3139

Key: NR Not recorded, VD Vaginal delivery, CS Caesarean section, PUV Pregnancy of unknown viability, LMP Last menstrual period, MSD Mean sac diameter, CRL Crown rump length, VIUP Viable intrauterine pregnancy.

### Automated segmentation model performance

Multi-task nnUNet (v2), a deep learning model designed for medical image segmentation, was used to automate the segmentation of the endometrial-myometrial border segment and the gestation sac segment. Performance was evaluated using the standard Dice Coefficient (DICE), measuring the pixel-overlap between predicted and ground-truth masks. The Deep Learning (DL) segmentation model for gestation sac achieved a mean standard DICE score of 0.950 and 0.940 in the training and test data sets respectively. The segmentation model for the sac endometrial border achieved a standard DICE mean score of 0.917 and 0.922 in the training and test data sets respectively.

**Fig. 3:**
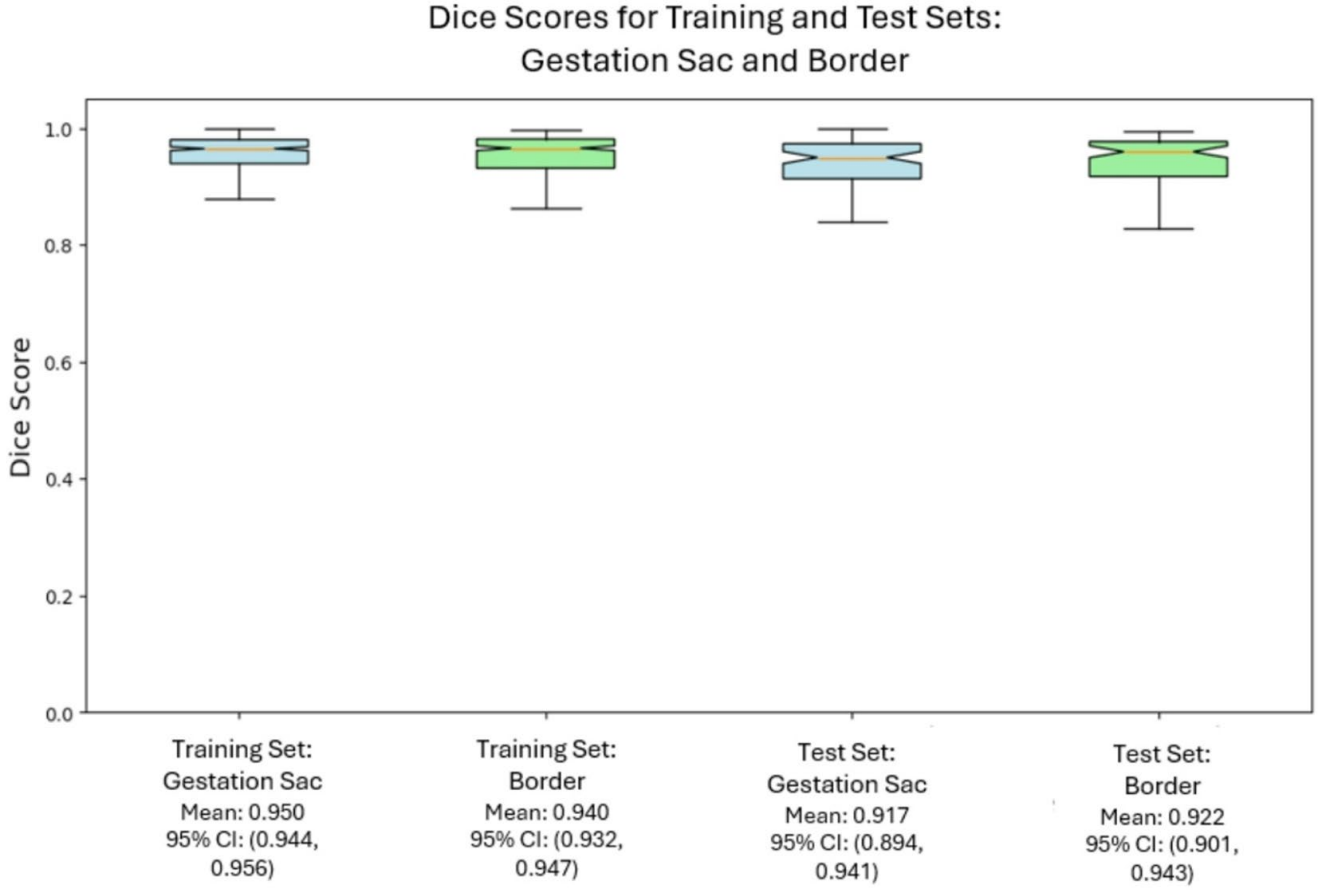
The performance of the Deep Learning (DL) segmentation models developed using Multi-task nnUNet (v2), compared to ground truth segmentation within QCCH training dataset and SMH validation dataset.

Figure 3 presents the performance of the Deep Learning (DL) segmentation models developed using Multi-task nnUNet (v2), compared to ground truth segmentation within QCCH training dataset and SMH validation dataset. The similarity scores (mean standard Dice Coefficient (DICE) scores), presented within a box plot, the middle line corresponds to the median, the upper and lower boundaries of the box correspond to upper and lower quartiles, whilst the whiskers reflect the minimum and maximum value and the white dots below the whiskers correspond to outliers.

**Fig. 4:**
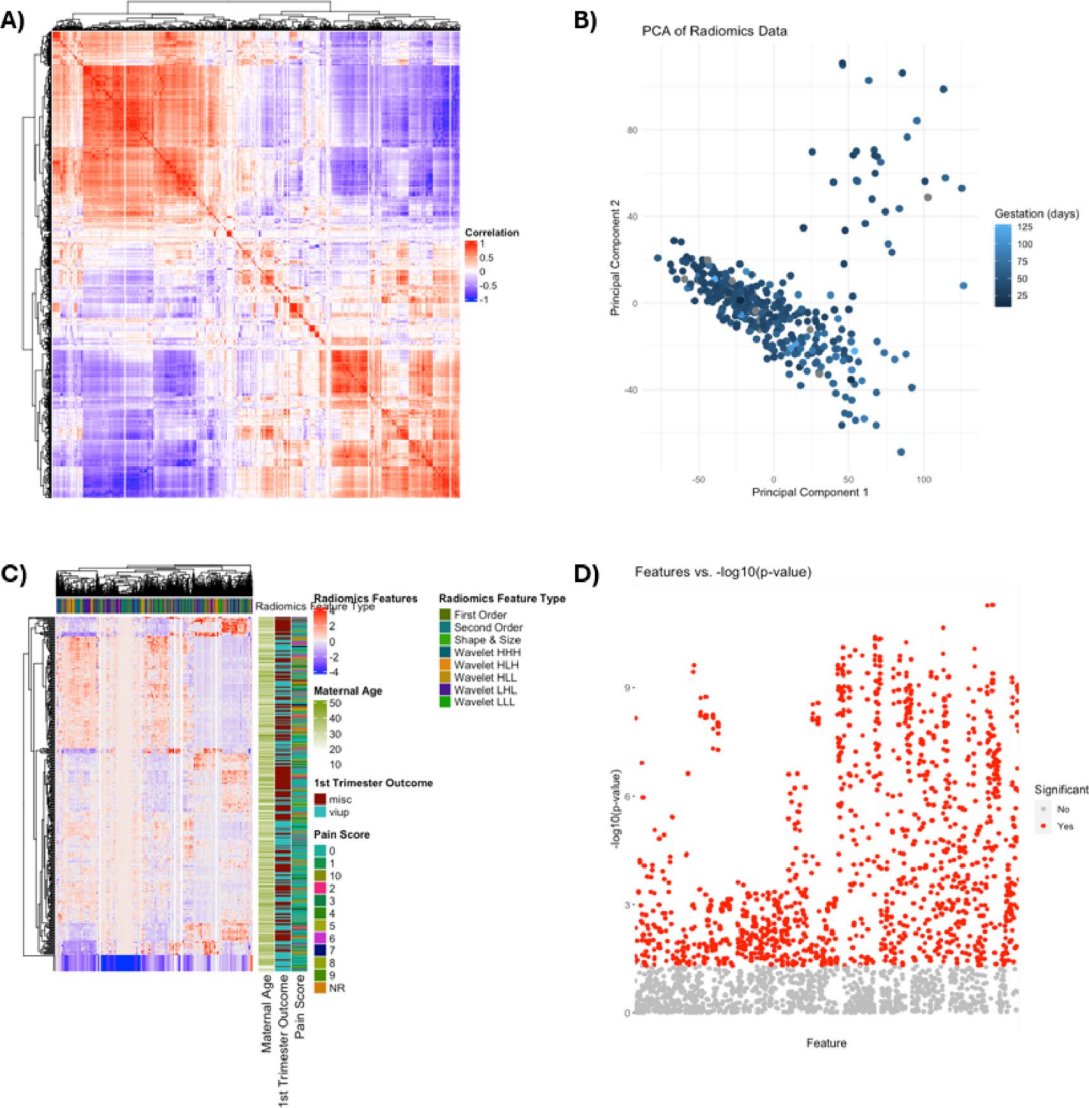
Overview of Radiomics Data Structure. Overview of Radiomics data structure. (**A**) Correlation matrix heat map of radiomics parameters. The degree of correlation between radiomics parameters is indicated within the heatmap (red indicates perfect correlation). (**B**) Principal component analysis (PCA) indicates the degree of variance within the dataset (**C**) Heatmap of all extracted radiomics features for early pregnancy outcome classification. Each row corresponds to an individual patient and each column corresponds to each scaled radiomics feature. The colour key outlines the corresponding radiomics feature sub-type. (**D**) Univariate logistic regression outlining radiomics features and their respective univariate logistic regression derived p values (horizontal red line indicates *p* < 0.01).

### Viability prediction model performance

The performance of radiomics features from both ROIs and the clinical data were analysed individually and then were combined, termed the Pregnancy of Unknown Viability Prediction Score (PUVPS). The results of individual analysis are included in the supplementary material.

**Fig. 5:**
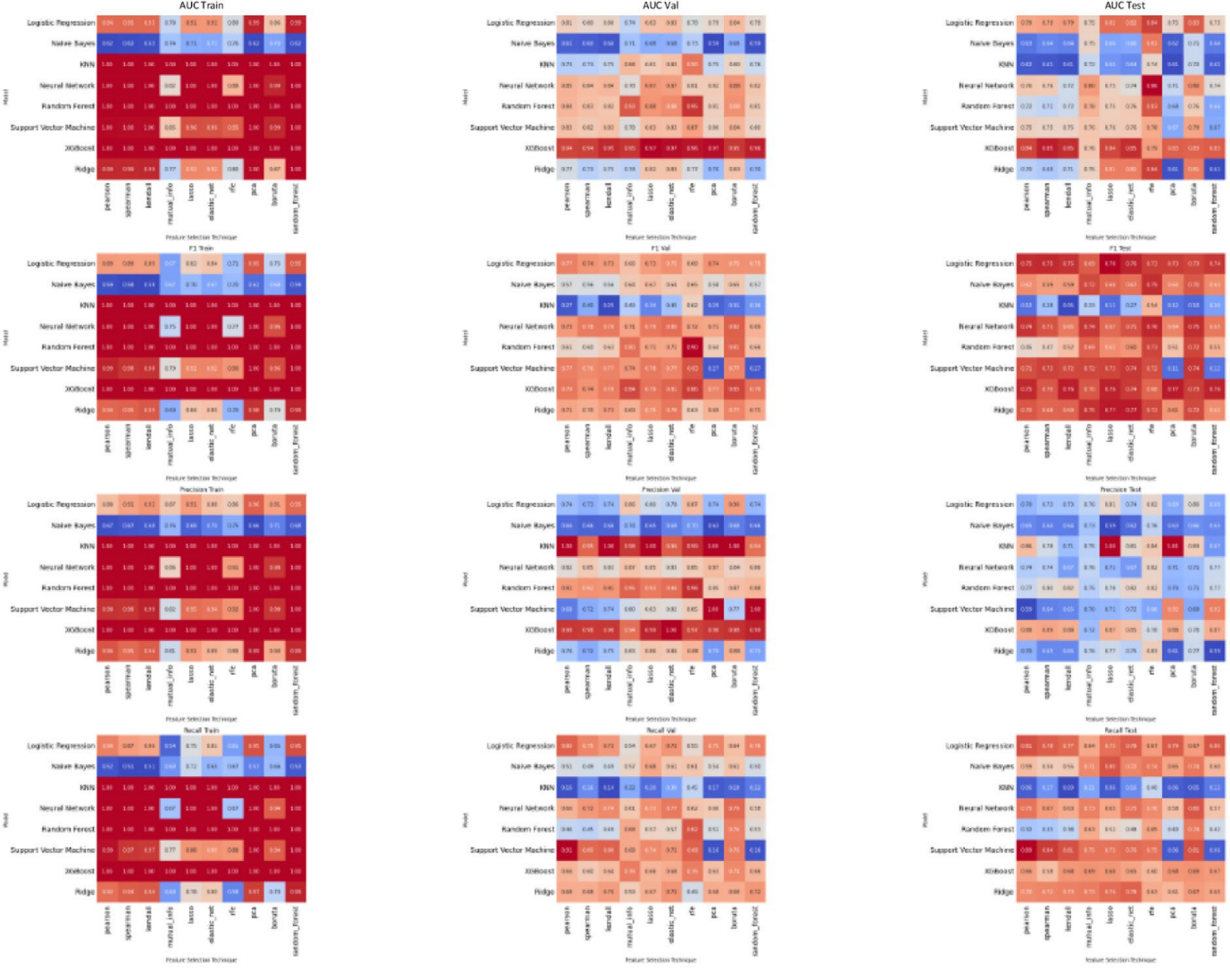
Clinical features and ultrasound radiomics combined: PUVPS model (Pregnancy of Unknown Viability Prediction Score Model).

**Fig. 6:**
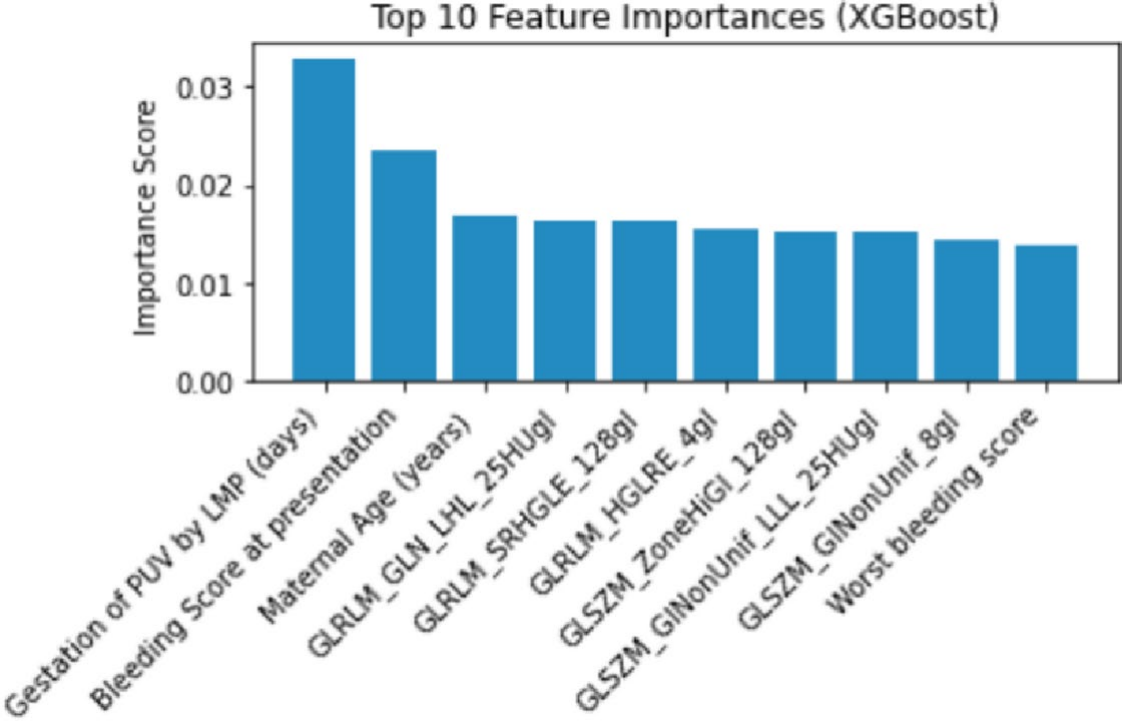
Feature Importance graph of XGBoost in the PUVPS model for prediction of viability.

Finally we combined the radiomic features of both analysed segments and the clinical features, to generate the Pregnancy of Unknown Viability Prediction Score (PUVPS). Figure 4 presents an overview of the radiomics data structure. The PUVPS model utilised XGBoost and LASSO feature selection technique (best performing identified by heatmaps Fig. 5), with AUC scores of 1.00 (F1-score 1.00), 0.92 (F1-score 0.79) and 0.84 (F1-score 0.76) in the QCCH training, QCCH validation and SMH test set respectively. The top ten features included in this model are presented in Fig. 6.

## Discussion

We have found that a model incorporating a combination of radiomics and clinical features can predict early pregnancy outcome with a good level of test performance (AUC 0.82). The PUVPS model is the first to evaluate early pregnancy ultrasound using an end-to-end method of segmentation and outcome prediction. This study is novel in assessing radiomic features to predict PUV outcome.

### Importance of radiomic features on predictive accuracy

The segmentation process involved thresholding to delineate the gestation sac from surrounding endometrial tissue, with expert review to confirm segmentation of the gestation sac and contents were achieved. Thus minimising subjective operator bias. Development of a segmentation model using deep learning, with test mean DICE scores of 0.940 and 0.922, will allow an end-to-end classification model that removes the need for expert clinician input to preprocessing of ultrasound images.

The methodical model development pipeline involved the evaluation of ten feature reduction and eight ML techniques in various combinations, to establish the best performing ML model (PUVPS model) for this outcome prediction task. The PUVPS model performed very well in both the QCCH (AUC 0.92) and SMH (AUC 0.84) validation datasets. Our results demonstrate the potential performance of an end-to-end model capable of stratifying risk of miscarriage for PUV.

Analysis of the radiomic features of high importance offers a degree of explainability of tissue biophysics to the model output. GLRLM (Gray-Level Run Length Matrix) features analyse the texture uniformity and consistency of the segmented ultrasound images. Higher non-uniformity suggests irregular tissue patterns linked to non-viable pregnancy. GLSZM (Gray-Level Size Zone Matrix) features measure the size and distribution of homogeneous intensity zones. An increase in non-uniformity and high intensity regions may indicate abnormal tissue structures, vascular abnormalities or poor placental development linked to increased likelihood of miscarriage.

Figure 4B is a PCA plot which visualises the relationship between radiomic features and gestational age using two principal components. A clear gradient is observed, where lighter blue points (higher GA) are more dispersed, whereas darker points (lower GA) are more clustered, indicating that radiomic features change with gestation. The data suggests that radiomics features change with gestational age and by analysing early pregnancy with more reliable gestational age, for example in in vitro fertilisation (IVF) pregnancy where embryo transfer date is known, specific radiomic features correlating with early pregnancy development may be identified in the future.

### Clinical features as complementary predictors

The feature with the highest importance score was ‘gestational age by LMP’, which is to be expected as delayed development with a PUV diagnosis on ultrasound relative to the expected gestational age likely indicates a delay in expected development, and thus a reduced chance of viability^14–16^. A quantitative bleeding score was the next most important feature, with the worst reported bleeding score also featuring in the top ten list, vaginal bleeding in early pregnancy is well documented a predictor of threatened miscarriage^14,17^. Maternal age was the third most important feature in the PUVPS model, with an increased maternal age associated with higher risk of aneuploidy and maternal co-morbidities, thus increasing the chance of first trimester miscarriage^18,19^.

### Model performance comparison

In the literature, the only previous model devised to predict PUV outcome based on clinical and ultrasound features is a logistic regression and scoring system to predict viability of a PUV, published by Bottomley et al., 2011^20^ and externally validated by Guha et al., 2013^21^ and Wan et al., 2020^22^.This model uses basic clinical and ultrasound features: maternal age, bleeding score, gestational age by last menstrual period (LMP), mean sac diameter and yolk sac presence. Cases where any of these variables were not documented or if LMP date was uncertain, were excluded. The output being a scoring system, demonstrating likelihood of viability at the end of the first trimester. The model performed with an AUC of 0.773 (95% CI 0.733–0.812) on the external validation data^21^. The advantage of the machine learning methods we have applied in deriving the best performing model is the applicability in cases where data points are missing.

There have been previous models which performed better, however these have required biochemical studies of blood samples from the pregnant women. Elson et al., 2003^23^ described a model which included serum progesterone measurement, and was validated by Lautmann et al., 2011^24^ with a AUC of 0.85. However, the validation study had issues with recruitment due to 90% women declining the extra blood test, which is not a routine part of clinical care in early pregnancy at the point of PUV diagnosis. It is a clear advantage, that the model we present does not require any further invasive tests and is based entirely on routinely collected clinical data in the early pregnancy setting.

Deep learning and radiomics have shown good model performance in differentiating malignant pathologies; in predicting lymph node metastasis in breast cancer^25^, classifying lymph nodes in lung cancer^26^, predicting human epidermal growth factor receptor 2 (HER2) status in breast cancer^27^ and classifying adnexal masses^13^. These models have AUC scores of 0.71^25^, 0.87^26^, 0.81^27^ and 0.90^13^, respectively. Radiomics has been explored in these oncology fields, yet here we present the first application to early pregnancy with good model performance.

### Implications for clinical practice

The value of a PUV outcome prediction model is in assessing risk, such a tool will allow patients and clinicians to make more informed decisions on monitoring and management. Ultimately, there will be no deviation from the protocol required to definitively diagnose miscarriage from a PUV, but this model can help women to navigate the uncertainty of a PUV diagnosis and equally support clinicians in advising patients in the 7/14 day wait for a repeat scan. Women with a high risk of miscarriage, may be counselled more closely on this, with information on what to expect and management options.

We know that early pregnancy loss is associated with significant psychological morbidity^1^. A study on the psychological impact of a PUV prediction scoring system by Lawson et al., 2023^28^, found that women reported that they found it helpful and 88.2% would opt to use it again, although assessment of psychological symptoms using the ANCOVA score did not show a statistical difference in anxiety, depression or worry between intervention groups using the prediction score and those in the control group. It is interesting to note that the reported patient experience, found that 69.1% agreed that the tool was useful to reduce the psychological impact of waiting to determine the outcome and 88.2% would chose to use the tool again^28^. The data supports patient value.

### Limitations and future directions

The main limitation of this study is its sample size, which can make an ML model prone to overfitting. This is demonstrated in the observed drop in classification performance and calibration of the PUVPS model between the QCCH validation and SMH test set. A future study could involve radiomic analysis of ultrasound images in a cohort of IVF pregnancies with a known embryo transfer date, to determine a clearer correlation between radiomic features and early pregnancy physiology. The next steps would be to establish a large multi-centre cohort to overcome the challenges associated with relatively small datasets, to validate across diverse populations to ensure reliability, thus improve the overall calibration of this model and potential clinical translation.

## Conclusion

Our study presents the development of a high-performing end-to-end ML model predicting early pregnancy PUV outcomes. With a larger data set to reduce over-fitting and wider external validation, we propose that this PUVPS model incorporating radiomics analysis of ultrasound image, alongside clinical features routinely collected, may serve to give women a personalised prediction of viability outcome. The capability to offer personalised outcome prediction, holds value in guiding clinician counselling and potentially improving the patient journey in navigating pregnancy uncertainty.

## Supplementary Information

Below is the link to the electronic supplementary material.

Supplementary Material 1

## Data Availability

The anonymised adnexal image datasets and corresponding clinical metadata used for model development and validation in this study are not publicly available due to privacy and ethical considerations. However, thesedatasets can be made accessible to qualified researchers upon reasonable request to the corresponding author.
