# Supplementary material for: Radiomics analysis of early pregnancy ultrasound images to predict viability at the end of first trimester
Source: Sci Rep. 2026 Jan 28;16:5504. doi: 10.1038/s41598-026-35158-5 (PMC12886957; doi:10.1038/s41598-026-35158-5)

**Supplementary Material**

The best performing model using radiomic analysis of the gestation sac border ROI (segment 1) utilised logistic regression with elastic net feature reduction, with an AUC scores of 0.90 in the QCCH training, 0.81 in QCCH validation and 0.65 in the SMH external test set (figure S.1.).

***Figure S.1. Gestation Sac Border (Segment 1) Radiomics Modelling for PUV Outcome Prediction.*** In heatmaps, x-axis corresponds to feature selection techniques and y-axis to modelling strategy. Upper row values are AUC scores and bottom row are F1 scores. Left to right are QCCH training, QCCH validation and SMH test set results, respectively.


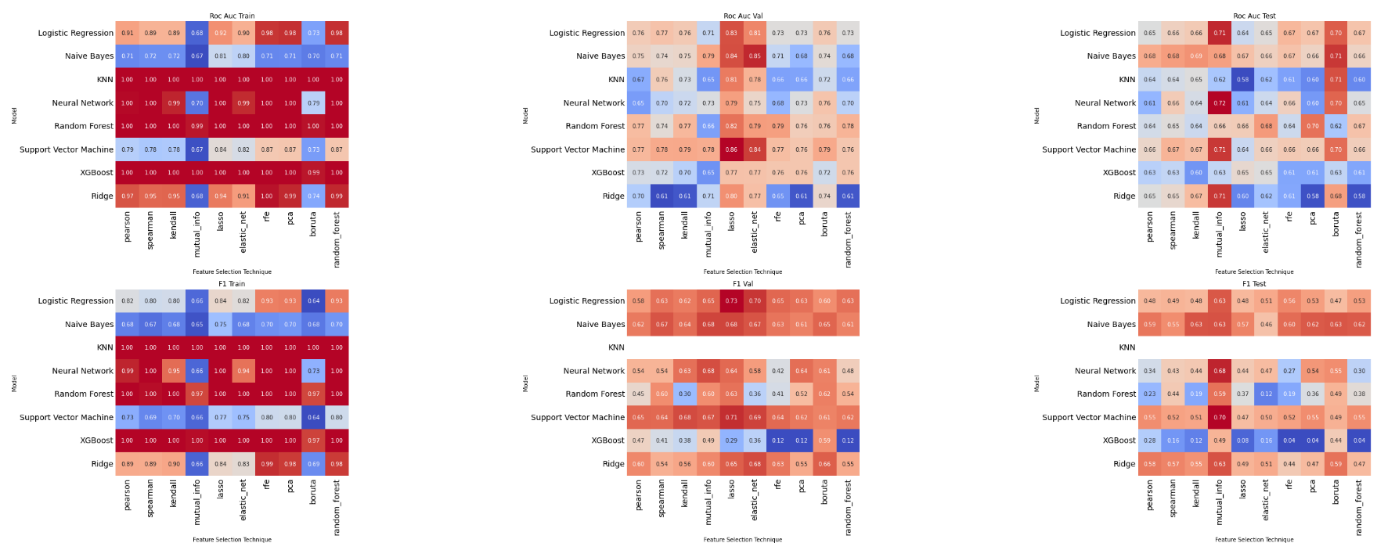


Radiomics analysis of the gestation sac and content ROI (segment 2) found the combination of Naïve Bayes algorithm and RFE feature reduction, resulted in the best performing model. AUC scores of 0.76 in the QCCH training, 0.78 in QCCH validation and 0.66 in the SMH external test set (Figure S.2.).

***Figure S.2. Gestation Sac and Contents (Segment 2) Radiomics Model for PUV Outcome Prediction.*** In heatmaps, x-axis corresponds to feature selection techniques and y-axis to modelling strategy. Upper row values are AUC scores and bottom row are F1 scores. Left to right are QCCH training, QCCH validation and SMH test set results, respectively.


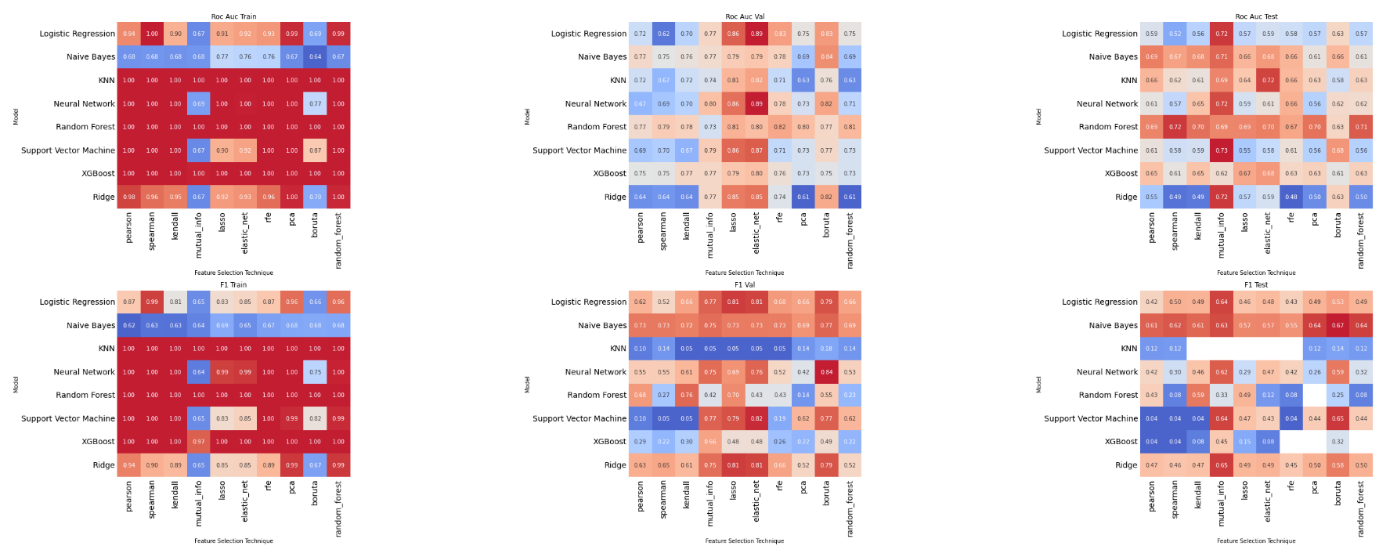


The best performing model using clinical variables for each of the PUV cases, culminated in a best performing model utilising logistic regression and RFE feature reduction methods. This combination generated AUC scores of 0.80 in the QCCH training, 0.85 in QCCH validation and 0.74 in the SMH external test set (Figure S.3.).

***Figure S.3. Clinical Features Model for PUV Outcome Prediction.*** In heatmaps, x-axis corresponds to feature selection techniques and y-axis to modelling strategy. Upper row values are AUC scores and bottom row are F1 scores. Left to right are QCCH training, QCCH validation and SMH test set results, respectively.


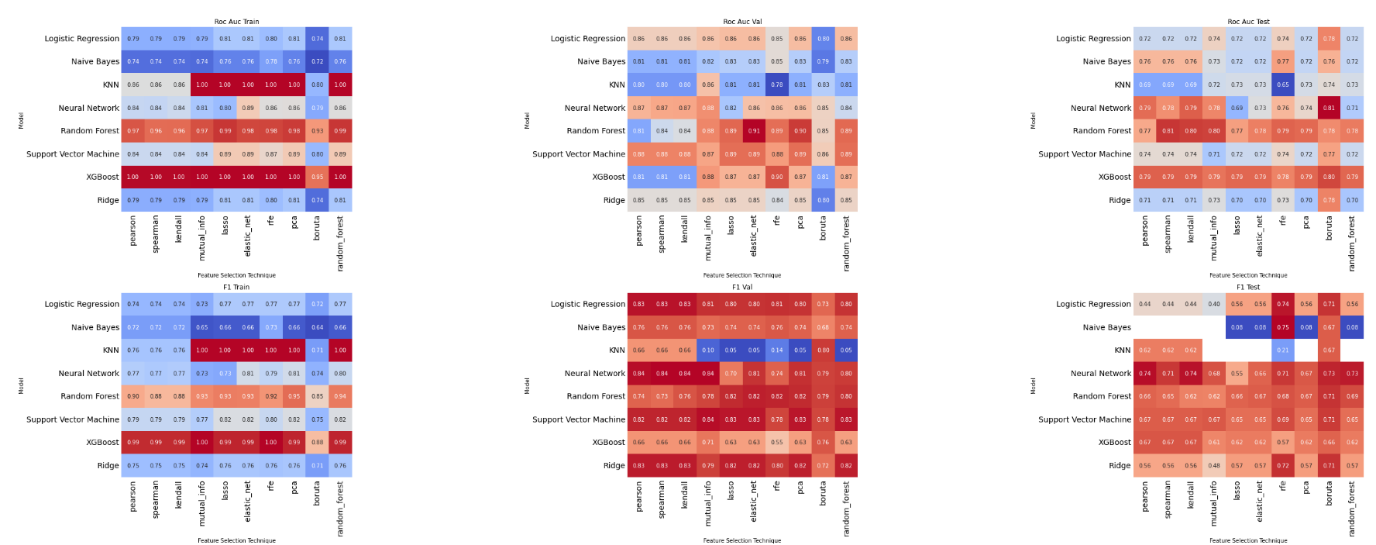

Supplement: Supplementary file 1 — Supplementary Material 1 [file 41598_2026_35158_MOESM1_ESM.docx]
